# Comparison of Copan ESwab and FLOQSwab for COVID-19 Diagnosis: Working around a Supply Shortage

**DOI:** 10.1128/JCM.00669-20

**Published:** 2020-05-26

**Authors:** Christie Vermeiren, Xavier Marchand-Senécal, Elena Sheldrake, David Bulir, Marek Smieja, Sylvia Chong, Jessica D. Forbes, Kevin Katz

**Affiliations:** a Shared Hospital Laboratory, Toronto, Ontario, Canada; b Department of Laboratory Medicine and Pathobiology, University of Toronto, Toronto, Ontario, Canada; c Sunnybrook Health Sciences Centre, Toronto, Ontario, Canada; d Department of Pathology and Molecular Medicine, McMaster University, Hamilton, Ontario, Canada; e Research Institute of St. Joe’s Hamilton, Hamilton, Ontario, Canada; Boston Children’s Hospital

**Keywords:** COVID-19, diagnostic microbiology, molecular diagnostic, public health

## LETTER

On 16 March 2020, the WHO Director-General stated, “You cannot fight a fire blindfolded. And we cannot stop this [COVID-19] pandemic if we don’t know who is infected. We have a simple message for all countries: test, test, test. Test every suspected case” (https://www.who.int/dg/speeches/detail/who-director-general-s-opening-remarks-at-the-media-briefing-on-covid-19---16-march-2020). This strategy hinges on the availability of appropriate, validated collection and transport systems to ensure preservation of nucleic acids and compatibility with downstream molecular testing—an acute challenge in the current pandemic. We present direct comparison of COVID-19 specimens collected with FLOQSwab nasopharyngeal swab preserved in universal transport medium (Copan UTM system; Copan, Italy; catalog no. 305C), optimized for viral specimens, and flocked regular nylon tip swab preserved in liquid Amies (ESwab collection system; Copan, Italy; catalog no. 480C), optimized for bacterial specimens.

COVID-19 symptomatic inpatients, outpatients, and emergency department patients across five hospitals were sampled with both collection systems. Nasopharyngeal sampling technique was used for the UTM collection system, and mid-turbinate sampling was used for the ESwab collection system. Paired specimens were sent to a centralized microbiology laboratory and processed using two distinct extraction/real-time reverse transcription-PCR (rRT-PCR) amplification platforms. In the first, nucleic acid extraction/amplification was performed on the BD Max System (Becton, Dickinson, USA), using the ExK TNA-2 extraction strip and detection of severe acute respiratory syndrome coronavirus 2 (SARS-CoV-2) 5′ untranslated region (UTR). Alternatively, specimens were extracted on the NucliSENS EasyMAG (bioMérieux, France) and detection of SARS-CoV-2 5′ UTR and envelope (1) was performed on the Rotor-gene Q (Qiagen, Germany). Both assays have been validated to detect 10 RNA copies/reaction (unpublished data).

**FIG 1:**
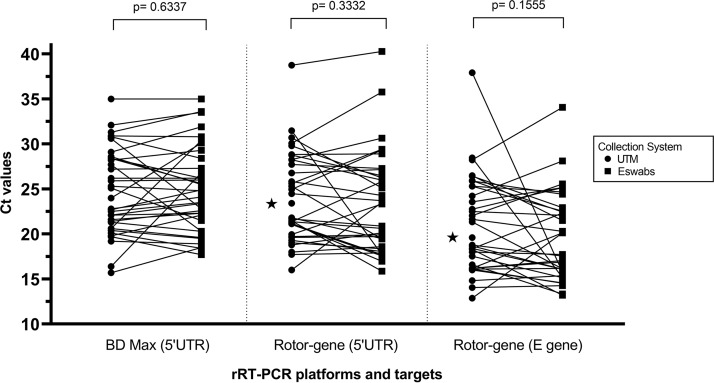
Diagram depicting UTM collection device and ESwab collection system paired *C_T_* values of positive SARS-CoV-2 detection for the different rRT-PCR targets and platforms. Values identified with a star represent discrepant qualitative results, where the rRT-PCR result of the other collection device is negative. A two-sided paired-sample *t* test found no statistically significant difference between the *C_T_* values, as shown by *P* values.

Paired specimens from 94 patients were analyzed. On the BD Max, 35 were concordantly positive and 59 were concordantly negative. There were no discrepant results. On the Rotor-gene, 1 pair of swabs could not be analyzed (disqualified), 33 were concordantly positive, 59 were concordantly negative, and 1 was only FLOQSwab positive. Positive and negative results were concordant between the 2 assays. Comparing swabs, positive percent agreement, negative percent agreement, and Cohen’s kappa values were 100% (95% confidence interval [CI], 0.900 to 1.000), 100% (95% CI, 0.939 to 1.000), and 1.00, respectively, on the BD Max and 97.1% (95% CI, 0.847 to 0.999), 100% (95% CI, 0.939 to 1.000), and 0.98, respectively, on the Rotor-gene. For positive swabs, the average cycle threshold (*C_T_*) values for each of the 3 rRT-PCR targets (5′ UTR in BD Max and 5′ UTR and envelope gene in Rotor-gene) did not show statistically significant difference with a 2-sided paired-sample *t* test between the 2 collection devices (Fig. 1).

To our knowledge, this is the first study comparing swabs and transport media for SARS-CoV-2 testing. Limitations include varied sampling technique, related to ESwabs being less flexible and therefore more difficult for reaching the nasopharynx. However, more-flexible ESwabs exist (catalog no. 482C). Second, detection of respiratory pathogens other than SARS-CoV-2 was not evaluated. Despite these limitations, this work clearly demonstrates, using two distinct downstream molecular testing methods, that the ESwab collection device is a suitable alternative to the UTM collection system in the context of an international swab shortage. We call upon the microbiology community to innovate and enhance testing capacity crucial to limit the spread of the current COVID-19 pandemic.
